# Properties of Residual Cumulative Sharma–Taneja–Mittal Model and Its Extensions in Reliability Theory with Applications to Human Health Analysis and Mixed Coherent Mechanisms

**DOI:** 10.3390/e28010032

**Published:** 2025-12-26

**Authors:** Mohamed Said Mohamed, Hanan H. Sakr

**Affiliations:** 1 Department of Mathematics, College of Science and Humanities, Prince Sattam bin Abdulaziz University, Hawtat Bani Tamim 16511, Saudi Arabia; mo.mahmoud@psau.edu.sa; 2 Department of Management Information Systems, College of Business Administration in Hawtat Bani Tamim, Prince Sattam bin Abdulaziz University, Al-Kharj 16273, Saudi Arabia

**Keywords:** function of mean life residual, non-parametric estimation, record statistics, residual cumulative entropy, stochastic comparison

## Abstract

The entropy measure of residual cumulative Sharma–Taneja–Mittal is an alternative measure of uncertainty for residual cumulative entropy. This study investigates further theoretical properties and develops nonparametric estimation procedures for the proposed measure. The performance of the estimator is evaluated through simulation experiments, and its practical relevance is illustrated using a real-world dataset on malignant tumor cases. Moreover, we investigate the properties of its dynamic version, including stochastic comparisons and its connections with the hazard rate function, mean residual function, and equilibrium random variables. Moreover, we introduce an alternative version of dynamic residual cumulative Sharma–Taneja–Mittal entropy and examine its monotonic properties. Additionally, we discuss this alternative version and its conditional form in the circumstances of record values. We introduce this alternative expression for the residual lifespan of upper record quantities in general distributions, characterizing it as a measure of upper record quantities derived from a distribution of uniform. Since Sharma–Taneja–Mittal entropy measures uncertainty, we also investigate its use in determining the entropy of the lifespan of mixed and coherent mechanisms, in which the lives of its constituent components are identically distributed and independent.

## 1. Introduction

Numerous disciplines, including the science of information, physics, statistical analysis, probability, theory of communications, and economics, have made extensive use of the entropy measure that Shannon [1] introduced. Assume that *Y* is the lifespan of a unit with a probability density function (pdf) k(y), a survival function $\bar{K}\left(y\right)=1-K\left(y\right)$, and a cumulative distribution function (cdf) K(y). If the expected quantity is present, the Shannon differential entropy of *Y*, which measures the uncertainty of a random phenomenon, is defined by

(1) $$\begin{array}{cc} \Lambda (Y) & =-E\left(\ln k\left(Y\right)\right)=-\int_{-\infty }^{\infty }k\left(y\right)\ln k\left(y\right)\;dy. \end{array}$$

The literature has presented a number of entropy measurements, each of which are appropriate for a particular set of circumstances. The uncertainty measure, known as the residual cumulative entropy, was proposed by Rao et al. [2] and is dependent on the cdf. The residual cumulative entropy for a non-negative continuously random variable *Y* with survival function $\bar{K}\left(y\right)$ is shown as

(2) $$\begin{array}{cc} R\Lambda (Y) & =-\int_{0}^{\infty }\bar{K}\left(y\right)\ln\bar{K}\left(y\right)\;dy. \end{array}$$

As noted by Asadi and Ebrahimi [3], Λ(Y) is no longer relevant for evaluating the uncertainty over the unit’s remaining lifespan if *Y* is taken to be the lifetime of a new unit. In these cases, the residual entropy measure of a component or system’s lifespan *Y* should be taken into account provided that the system has lived to an age *l*. The dynamical or dependent on time random variable $Y_{l}=\left(Y-l|Y>l\right)$ with a survival function is what we are interested in examining in these situations:

$${\bar{K}}_{l}\left(y\right)=\left\{\begin{array}{cc} \frac{\bar{K}\left(y\right)}{\bar{K}\left(l\right)}, & \mathrm{if}\;y>l,\\ 1, & \mathrm{otherwise}, \end{array}\right.$$

where y>l, and both *y* and *l* are positive. Then the function of mean life residual (MHr(l)) is defined as $MHr\left(l\right)=E\left(Y_{l}\right)=\int_{l}^{\infty }\frac{\bar{K}\left(y\right)}{\bar{K}\left(l\right)}\;dy$. Asadi and Zohrevand [4] have adjusted the concept of residual cumulative entropy to account for the system’s present age by using the survival function of $Y_{l}$ as

(3) $$\begin{array}{cc} DR\Lambda (Y;l) & =-\int_{l}^{\infty }\frac{\bar{K}\left(y\right)}{\bar{K}\left(l\right)}\ln\frac{\bar{K}\left(y\right)}{\bar{K}\left(l\right)}\;dy. \end{array}$$

The measure DRΛ(Y;l) is known as the dynamic residual cumulative entropy measure. DRΛ(Y;0)=RΛ(Y) is evident. Address Rao et al. [2], Asadi and Zohrevand [4], Navarro et al. [5], and the articles cited therein for more characteristics and uses of (2) and (3).

Another alternative form of the dynamic residual cumulative entropy measure can be obtained by considering the pdf of $Y_{l}$, symbolized as $k_{l}\left(y\right)=\frac{k(y+l)}{\bar{K}\left(l\right)}$, in which *y* and *l* are both positive. Ebrahimi [6] proposed this measure of $Y_{l}$ as

(4) $$\begin{array}{cc} DR\Lambda^{*}\left(Y;l\right) & =-\int_{l}^{\infty }\frac{k(y)}{\bar{K}\left(l\right)}\ln\frac{k(y)}{\bar{K}\left(l\right)}\;dy. \end{array}$$

In addition to other studies cited in their work, several academics, including Asadi et al. [7], Nanda and Paul [8], and Zhang [9], have conducted extensive research on the properties, extensions, and applications of $DR\Lambda^{*}\left(Y;l\right)$.

Another interesting measure of uncertainty is the Sharma–Taneja–Mittal entropy, independently introduced by Sharma and Taneja [10] and by Mittal [11] and defined as follows:

(5) $$\begin{array}{cc} S\Lambda \tau_{1},\tau_{2}\left(Y\right) & =\frac{1}{\tau_{1}-\tau_{2}}\int_{0}^{\infty }\left(k^{\tau_{1}}\left(y\right)-k^{\tau_{2}}\left(y\right)\right)dy\\ & =\frac{1}{\tau_{1}-\tau_{2}}\left[E\left(k^{\tau_{1}-1}\left(K^{-1}\left(V\right)\right)\right)-E\left(k^{\tau_{2}-1}\left(K^{-1}\left(V\right)\right)\right)\right], \end{array}$$

with the condition that $\tau_{1}\neq \tau_{2}>0$, and $K^{-1}\left(v\right)=\inf\;\left\{y:K\left(y\right)\geq v\right\}$, v∈[0,1] stands for the quantile function. Moreover, the Sharma–Taneja–Mittal entropy measure provided in (5) could be considered a further development of negative Tsallis entropy if $\tau_{2}=1$, see Tsallis [12].

More recently, Kattumannil et al. [13] proposed definitions for the Sharma–Taneja–Mittal residual cumulative entropy and cumulative entropy, given by

(6) $$\begin{array}{c} RS\Lambda \tau_{1},\tau_{2}\left(Y\right)=\frac{1}{\tau_{1}-\tau_{2}}\int_{0}^{\infty }\left({\bar{K}}^{\tau_{1}}\left(y\right)-{\bar{K}}^{\tau_{2}}\left(y\right)\right)dy, \end{array}$$

(7) $$\begin{array}{c} CS\Lambda \tau_{1},\tau_{2}\left(Y\right)=\frac{1}{\tau_{1}-\tau_{2}}\int_{0}^{\infty }\left(K^{\tau_{1}}\left(y\right)-K^{\tau_{2}}\left(y\right)\right)dy, \end{array}$$

again under the restriction that $\tau_{1}\neq \tau_{2}>0$. Subsequently, Sudheesh et al. [14] reformulated the measures in the above expressions in terms of probability-weighted moments, thereby paving the way for the development of inferential techniques based on the established properties of these moments. Moreover, Sakr and Mohamed [15] discussed the Sharma–Taneja–Mittal entropy measure in estimation and goodness-of-fit test. In addition, several works have explored the Sharma–Mittal framework and its related extensions in diverse situations. For instance, Ghaffari et al. [16] examined thermodynamic characteristics of Schwarzschild black holes within the Sharma–Mittal entropy formalism. Koltcov et al. [17] employed the Sharma–Mittal entropy to assess the performance of topic modeling techniques. In the setting of record values, Paul and Thomas [18] investigated key properties associated with the Sharma–Mittal entropy. More recently, Sfetcu et al. [19] introduced an alternative form of the cumulative residual Sharma–Taneja–Mittal entropy and established corresponding upper and lower bounds.

This paper discusses the properties of Sharma–Taneja–Mittal entropy and its extensions. The organization of the rest of the article is as follows: Section 2 presents the proportional hazard framework of the residual cumulative Sharma–Taneja–Mittal entropy and its non-parametric estimation using a U-statistic. Section 3 explores the properties of the dynamic residual cumulative Sharma–Taneja–Mittal entropy, including stochastic comparisons and their connections to hazard rate functions, mean residual functions, and equilibrium random variables. Section 4 introduces an alternative dynamic form of the residual cumulative Sharma–Taneja–Mittal entropy and examines its properties with ordered variables. Finally, Section 5 presents the Sharma–Taneja–Mittal entropy and its properties for coherent and mixed structures in independent and identically distributed situations.

## 2. Properties of Residual Cumulative Sharma–Taneja–Mittal Entropy

In this section, we will discuss some further properties of the residual cumulative Sharma–Taneja–Mittal entropy introduced in (6). We demonstrate that $RS\Lambda \tau_{1},\tau_{2}\left(Y\right)$ is shifting as an independent measure in the following property.

**Theorem** **1.** *Let Z=αY+β, α>0, β≥0, and let Y be a continuous non-negative random variable. Next,*

$$RS\Lambda \tau_{1},\tau_{2}\left(Z\right)=\alpha \cdot RS\Lambda \tau_{1},\tau_{2}\left(Y\right).$$

**Proof.**  The conclusion is that for every y>β, ${\bar{K}}_{\alpha Y+\beta }\left(y\right)={\bar{K}}_{Y}\left(\frac{y-\beta }{\alpha }\right)$. □

**Theorem** **2.** *Assume that two random variables, Y and $Z_{\eta }$, admit a proportional hazard framework, provided by*

$${\bar{K}}_{\eta }^{*}\left(y\right)={\left(\bar{K}\left(y\right)\right)}^{\eta },\;\eta >0,\;y>0.$$

*Then the following claims are true:*

(*i*) *$RS\Lambda \tau_{1},\tau_{2}\left(Z_{\eta }\right)=RS\Lambda \eta \tau_{1},\eta \tau_{2}\left(Y\right)$,*
(*ii*) *$RS\Lambda \tau_{1},\tau_{2}\left(Z_{\eta }\right)=\eta \cdot RS\Lambda \tau_{1},\tau_{2}\left(\eta Y\right)$.*

**Corollary** **1.** *Suppose X becomes a random variable that is not negative and has a cdf of K that is absolutely continuous. Using a random sample of K, let $Y_{1:p}$ be the first-order statistic based on $Y_{1},Y_{2},\ldots ,Y_{p}$. We know ${\bar{K}}_{Y_{1:p}}\left(y\right)={\left(\bar{K}\left(y\right)\right)}^{p}$, and so $RS\Lambda \tau_{1},\tau_{2}\left(Y_{1:p}\right)=RS\Lambda p\tau_{1},p\tau_{2}\left(Y\right)$.*

**Example** **1.** *Suppose the random variable Y follows an exponential distribution with parameter θ, i.e.,Y∼Expo(θ), then $RS\Lambda \tau_{1},\tau_{2}\left(Z_{\eta }\right)=\frac{1}{\tau_{1}-\tau_{2}}\left(\frac{1}{\theta \eta \tau_{1}}-\frac{1}{\theta \eta \tau_{2}}\right)$, $RS\Lambda \eta \tau_{1},\eta \tau_{2}\left(Y\right)=\frac{1}{\tau_{1}-\tau_{2}}\left(\frac{1}{\theta \eta \tau_{1}}-\frac{1}{\theta \eta \tau_{2}}\right)$, and $RS\Lambda \tau_{1},\tau_{2}\left(Y\right)=\frac{1}{\tau_{1}-\tau_{2}}\left(\frac{1}{\theta \tau_{1}}-\frac{1}{\theta \tau_{2}}\right)$. So, we have $RS\Lambda \tau_{1},\tau_{2}\left(Z_{\eta }\right)=RS\Lambda \eta \tau_{1},\eta \tau_{2}\left(Y\right)\;\mathrm{and}\;RS\Lambda \tau_{1},\tau_{2}\left(Y\right)=\eta \cdot RS\Lambda \eta \tau_{1},\eta \tau_{2}\left(Y\right)$.*

We now derive an estimator for $RS\Lambda_{\tau_{1},\tau_{2}}\left(Y\right)$. Let $\tau_{1}\neq \tau_{2}>0$, and assume that $Y_{1:p}$ represents the smallest order statistic from a random sample $Y_{1},\ldots ,Y_{p}$ drawn from the distribution *F*. For a non-negative random variable *Y*, its expectation is given by $\mu =E\left(Y\right)=\int_{0}^{\infty }\bar{K}\left(y\right)\;dy$. Thus, we can express

$$\begin{array}{cc} RS\Lambda_{\tau_{1},\tau_{2}}\left(Y\right) & =\frac{1}{\tau_{1}-\tau_{2}}\left(\int_{0}^{\infty }{\bar{K}}^{\tau_{1}}\left(y\right)\;dy-\int_{0}^{\infty }{\bar{K}}^{\tau_{2}}\left(y\right)\;dy\right)\\ & =\frac{1}{\tau_{1}-\tau_{2}}\left(E\left(Y_{1:\tau_{1}}\right)-E\left(Y_{1:\tau_{2}}\right)\right). \end{array}$$

This motivates the construction of a U-statistic–based estimator for $RS\Lambda_{\tau_{1},\tau_{2}}\left(Y\right)$ as

(8) $${R\hat{S}\Lambda }_{\tau_{1},\tau_{2}}\left(Y\right)=\frac{1}{\tau_{1}-\tau_{2}}\left(\frac{1}{A_{\tau_{1},p}}\sum_{A_{\tau_{1},p}}\min\left(Y_{j_{1}},Y_{j_{2}},\ldots ,Y_{j_{\tau_{1}}}\right)-\frac{1}{A_{\tau_{2},p}}\sum_{A_{\tau_{2},p}}\min\left(Y_{j_{1}},Y_{j_{2}},\ldots ,Y_{j_{\tau_{2}}}\right)\right),$$

where the summations extend over the set $A_{\tau_{i},p}$ containing all combinations of $\tau_{i}$ distinct elements $\left\{Y_{j_{1}},Y_{j_{2}},\ldots ,Y_{j_{\tau_{i}}}\right\}$ selected from {1,2,…,p} for i=1,2.

Next, we simplify the expression for ${R\hat{S}\Lambda }_{\tau_{1},\tau_{2}}\left(Y\right)$. Denote by $Y_{j:p}$ the *j*-th order statistic based on the sample $Y_{1},\ldots ,Y_{p}$ drawn from *K*. In terms of these order statistics, the following equivalent formulations hold:

$$\sum_{j=1}^{p}\sum_{\begin{array}{c} s=1\\ s<j \end{array}}^{p}\min\left\{Y_{1},Y_{2}\right\}=\sum_{j=1}^{p}\left(p-j\right)Y_{1:p},$$

and

∑j=1p∑s=1s<jp∑r=1r<spmin{Y1,Y2,Y3}=∑j=1p(p−j−1)(p−j)Y1:p =∑j=1pp−2jY1:p.

Thus, the estimator in (8) can be alternatively written as

(9) RS^Λτ1,τ2(Y)=1τ1−τ21Aτ1,p∑j=1pp−jτ1−1Y1:p−1Aτ2,p∑j=1pp−jτ2−1Y1:p.

We now examine the asymptotic properties of ${R\hat{S}\Lambda }_{\tau_{1},\tau_{2}}\left(Y\right)$. It is evident that this estimator is both unbiased and consistent for $C_{s}\left(X\right)$ (see Lehmann [20]). The following theorem characterizes its asymptotic distribution.

**Theorem** **3.** *As p→∞, the scaled difference*

$$\sqrt{p}\left({R\hat{S}\Lambda }_{\tau_{1},\tau_{2}}\left(Y\right)-RS\Lambda_{\tau_{1},\tau_{2}}\left(Y\right)\right)$$

*converges in distribution to a normal random variable with mean zero and variance*

(10) $$\begin{array}{c} \sigma^{2}=\frac{1}{{\left(\tau_{1}-\tau_{2}\right)}^{2}}\;Var\left[\left(Y\;{\bar{K}}^{\tau_{1}-1}\left(Y\right)+\left(\tau_{1}-1\right)\int_{0}^{Y}z\;{\bar{K}}^{\tau_{1}-2}\left(z\right)\;dK\left(z\right)\right)\right.\\ -\left.\left(Y\;{\bar{K}}^{\tau_{2}-1}\left(Y\right)+\left(\tau_{2}-1\right)\int_{0}^{Y}z\;{\bar{K}}^{\tau_{2}-2}\left(z\right)\;dK\left(z\right)\right)\right]. \end{array}$$

**Proof.**  By employing the central limit theorem for U-statistics, the asymptotic normality of ${R\hat{S}\Lambda }_{\tau_{1},\tau_{2}}\left(Y\right)$ follows. The asymptotic variance, denoted $\sigma_{1}^{2}$, is provided by Lee [21] as

(11) $$\sigma_{1}^{2}=\frac{1}{{\left(\tau_{1}-\tau_{2}\right)}^{2}}\;Var\left[E\left(\min\left(Y_{2},\ldots ,Y_{\tau_{1}}\right)|Y_{1}\right)-E\left(\min\left(Y_{2},\ldots ,Y_{\tau_{2}}\right)|Y_{1}\right)\right].$$

Define $Z=\min(Y_{2},Y_{3},\ldots ,Y_{\tau_{i}})$; then, its survival function is ${\bar{K}}^{\tau_{i}-1}\left(y\right)$ for i=1,2. Notice that

$$\begin{array}{cc} E\left[\min(y,Y_{2},Y_{3},\ldots ,Y_{\tau_{i}})\right] & =E\left[y\;1\left(Z>y\right)\right]+E\left[Z\;1(Z\leq y)\right]\\ & =y\;{\bar{K}}^{\tau_{i}-1}\left(y\right)+\left(\tau_{i}-1\right)\int_{0}^{y}z\;{\bar{K}}^{\tau_{i}-2}\left(z\right)dF\left(z\right). \end{array}$$

Substituting this result into (11) yields the variance expression shown in (10), which completes the proof. □

Finally, the finite-sample performance of the estimator in (9) is assessed via Monte Carlo simulations, with the results detailed in the next subsection.

### 2.1. Simulation Studies

Using the exponential distribution with rate 0.5 and the gamma distribution with shape parameter equal 2 and scale parameter equal 2 (Gamma(2,2)), we perform a comprehensive Monte Carlo simulation research to evaluate the estimator performance of the suggested measure. Using R software (version 4.4.1), the simulation is run ten thousand times with varying sample sizes. Table 1 and Table 2 present the theoretical value of the residual cumulative Sharma–Taneja–Mittal entropy as well as the variance and the mean square error root (MSER) of the estimator ${R\hat{S}\Lambda }_{\tau_{1},\tau_{2}}\left(Y\right)$ with different values of $\tau_{1}$ and $\tau_{2}$. The results in Table 1 and Table 2 indicate that for a fixed pair $\left(\tau_{1},\tau_{2}\right)$, increasing the dimension *p* significantly reduces both the variance and the MSER of the residual cumulative Sharma–Taneja–Mittal entropy estimator, demonstrating enhanced estimation accuracy with larger *p*. Moreover, as the difference between $\tau_{1}$ and $\tau_{2}$ increases—reflected by the $RS\Lambda_{\tau_{1},\tau_{2}}\left(Y\right)$ index becoming less negative—the estimator exhibits further improvements, with lower variance and MSER observed. This suggests that a larger disparity between the tuning parameters, in addition to an increased *p*, contributes to a more robust and efficient estimation by better balancing bias and variance in the estimation procedure.

**Figure 1:**
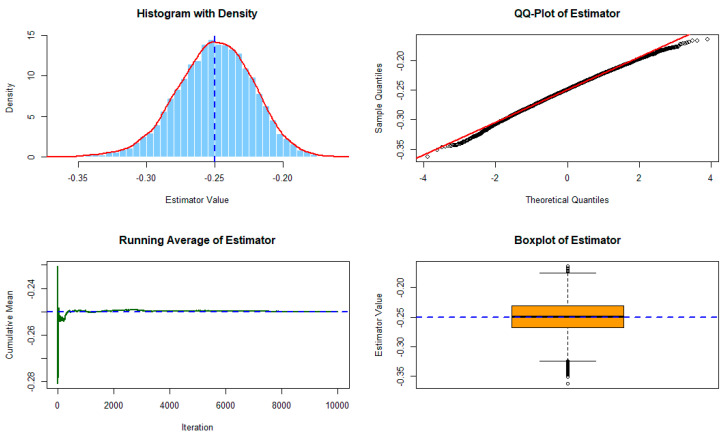
Performance of the residual cumulative Sharma–Taneja–Mittal entropy estimator from 10,000 simulations using an exponential distribution with rate 0.5. Panels: (TL) Histogram with density and theoretical value (−0.25); (TR) QQ-plot; (BL) Running average; (BR) Boxplot.

**Figure 2:**
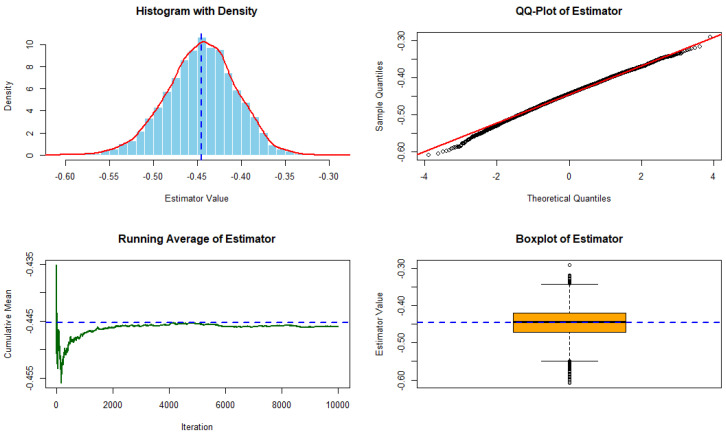
Performance of the residual cumulative Sharma–Taneja–Mittal entropy estimator from 10,000 simulations using Gamma(2,2) distribution. Panels: (TL) Histogram with density and theoretical value (−0.445313); (TR) QQ-plot; (BL) Running average; (BR) Boxplot.

Under $\tau_{1}=2$, $\tau_{2}=4$, and p=100, Figure 1 and Figure 2 show the performance of the residual cumulative Sharma–Taneja–Mittal entropy estimator computed from 10,000 simulations using an exponential distribution with rate 0.5 and Gamma(2,2) distribution, respectively. The top left (TL) panel displays the histogram with a kernel density overlay and the theoretical value marked by a dashed line. The top right (TR) panel shows the QQ-plot for assessing normality. The bottom left (BL) panel illustrates the running cumulative average, demonstrating convergence to the theoretical value, while the bottom right (BR) panel presents a boxplot summarizing the estimator’s dispersion.

### 2.2. Real Data Application

In this subsection, a real dataset is analyzed to illustrate the practical applicability of the proposed methodology. The dataset comprises records of malignant tumor cases collected from King Faisal Specialist Hospital and Research Centre, classified by tumor site and gender for the year 2023 in Saudi Arabia. The analysis is restricted to female patients from the Jeddah region. This dataset is publicly accessible via the Saudi Open Data Portal at https://open.data.gov.sa/ar/datasets/view/190d7539-ba90-47f2-a162-f2b64b3c41d1 (accessed on 5 November 2025).

The observations were modeled using an exponential distribution whose cdf is expressed as $G\left(y\right)=1-e^{-\theta y},\;\theta >0,\;y\geq 0$. Parameter estimation was carried out using the maximum likelihood estimation method, resulting in an estimated rate parameter of θ=0.01798561. The goodness-of-fit of the model was assessed through two statistical tests:

1. Kolmogorov–Smirnov test: statistic = 0.29587, with *p*-value = 0.1168;
2. Anderson–Darling test: statistic = 2.471, with *p*-value = 0.05213.

**Figure 3:**
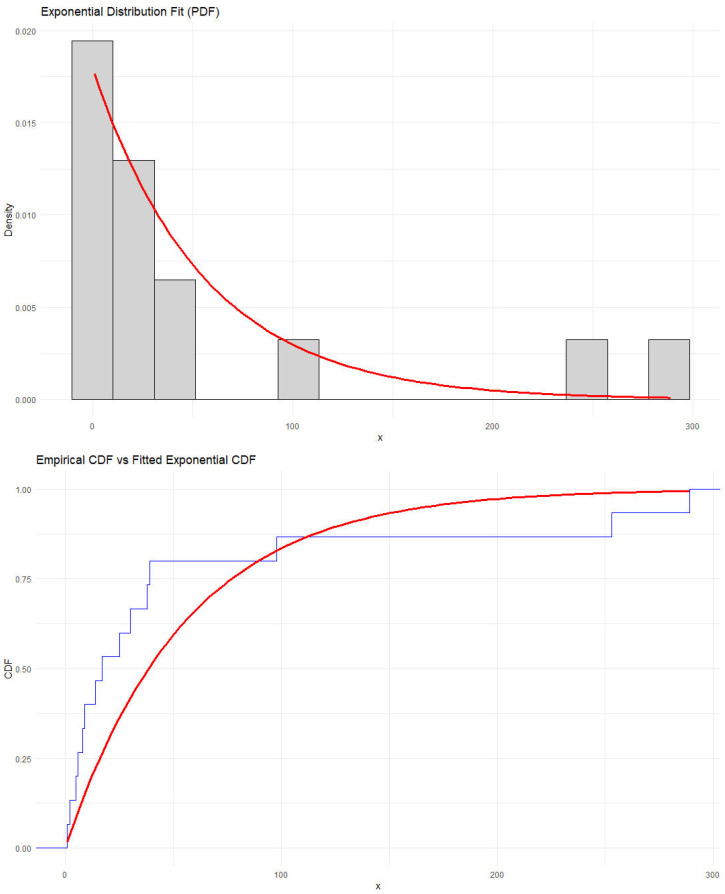
Histogram with fitted pdf (**up**) and Empirical cdf vs. fitted cdf (**down**).

Both tests support the adequacy of the exponential model in describing the observed data. Additionally, Figure 3 presents the histogram of the data along with the fitted pdf and a comparison between the empirical and fitted cdfs.

We run 10,000 bootstrap samples to examine the estimate for the real set of data. Table 3 shows the bootstrap estimates of the proposed estimator for several combinations of $\left(\tau_{1},\tau_{2}\right)$ based on 10,000 resamples from the observed data. The results indicate that the bootstrap mean values are very close to the original estimates across all combinations, suggesting that the estimator is stable and exhibits only minor bias. The standard deviations and the associated 95% confidence intervals confirm that the variability of the estimator decreases as the values of $\left(\tau_{1},\tau_{2}\right)$ increase, reflecting improved precision for larger order parameters. Overall, the bootstrap analysis supports the consistency and robustness of the proposed estimator under repeated sampling.

## 3. Dynamic Residual Cumulative Sharma–Taneja–Mittal Entropy

In this section, we will define the dynamic residual cumulative Sharma–Taneja–Mittal entropy. In analogue to (3), we can define the dynamic residual cumulative Sharma–Taneja–Mittal entropy as

(12) $$\begin{array}{cc} DRS\Lambda_{\tau_{1},\tau_{2}}\left(Y;l\right) & =\frac{1}{\tau_{1}-\tau_{2}}\left[\int_{l}^{\infty }{\left(\frac{\bar{K}\left(y\right)}{\bar{K}\left(l\right)}\right)}^{\tau_{1}}\;dy-\int_{l}^{\infty }{\left(\frac{\bar{K}\left(y\right)}{\bar{K}\left(l\right)}\right)}^{\tau_{1}}\;dy\right], \end{array}$$

with the condition that $\tau_{1}\neq \tau_{2}>0$, and we readily recognize that $DRS\Lambda_{\tau_{1},\tau_{2}}\left(Y;0\right)=RS\Lambda_{\tau_{1},\tau_{2}}\left(Y\right)$, at l=0.

**Proposition** **1.** *$DRS\Lambda_{\tau_{1},\tau_{2}}\left(Y;l\right)$ is always non-positive.*

**Proof.**  For $\tau_{1}\geq \left(\leq \right)\tau_{2}$, we have ${\left(\frac{\bar{K}\left(y\right)}{\bar{K}\left(l\right)}\right)}^{\tau_{1}}\leq \left(\geq \right){\left(\frac{\bar{K}\left(y\right)}{\bar{K}\left(l\right)}\right)}^{\tau_{2}}$, y≥l, which proves the result. □

**Lemma** **1.** *In the event when Z=ψ(Y) is an increasing differentiated function, the random variable Z’s dynamic residual cumulative Sharma–Taneja–Mittal entropy can be found using*

(13) $$\begin{array}{cc} DRS\Lambda_{\tau_{1},\tau_{2}}\left(Z;l\right) & =\frac{1}{\tau_{1}-\tau_{2}}\left[\frac{1}{\left({\bar{K}}^{\tau_{1}}\left(\psi^{-1}\left(l\right)\right)\right)}\int_{\psi^{-1}\left(l\right)}^{\infty }{\bar{K}}^{\tau_{1}}\left(y\right)\psi^{\prime }\left(y\right)\;dy\right.\\ & \;-\left.\frac{1}{\left({\bar{K}}^{\tau_{2}}\left(\psi^{-1}\left(l\right)\right)\right)}\int_{\psi^{-1}\left(l\right)}^{\infty }{\bar{K}}^{\tau_{2}}\left(y\right)\psi^{\prime }\left(y\right)\;dy\right]. \end{array}$$

**Example** **2.** *Let Z=αY+β for a non-negative randomly selected variable Y, where α>0 and β≥0. Next, we have*

$$\begin{array}{cc} DRS\Lambda_{\tau_{1},\tau_{2}}\left(Z;l\right) & =\frac{1}{\tau_{1}-\tau_{2}}\left[\frac{1}{\left({\bar{K}}^{\tau_{1}}\left(\frac{l-\beta }{\alpha }\right)\right)}\int_{l}^{\infty }{\bar{K}}^{\tau_{1}}\left(\frac{y-\beta }{\alpha }\right)\psi^{\prime }\left(y\right)\;dy\right.\\ & \;-\left.\frac{1}{\left({\bar{K}}^{\tau_{2}}\left(\frac{l-\beta }{\alpha }\right)\right)}\int_{l}^{\infty }{\bar{K}}^{\tau_{2}}\left(\frac{y-\beta }{\alpha }\right)\psi^{\prime }\left(y\right)\;dy\right]\\ & =\frac{\alpha }{\tau_{1}-\tau_{2}}\left[\frac{1}{\left({\bar{K}}^{\tau_{1}}\left(\frac{l-\beta }{\alpha }\right)\right)}\int_{\frac{l-\beta }{\alpha }}^{\infty }{\bar{K}}^{\tau_{1}}\left(v\right)\;dv\right.-\left.\frac{1}{\left({\bar{K}}^{\tau_{2}}\left(\frac{l-\beta }{\alpha }\right)\right)}\int_{\frac{l-\beta }{\alpha }}^{\infty }{\bar{K}}^{\tau_{2}}\left(v\right)\;dv\right]\\ & =\alpha DRS\Lambda_{\tau_{1},\tau_{2}}\left(Y;\frac{l-\beta }{\alpha }\right). \end{array}$$

*Moreover, we can see that*

*1*. *$DRS\Lambda_{\tau_{1},\tau_{2}}\left(\alpha Y;l\right)=\alpha \;DRS\Lambda_{\tau_{1},\tau_{2}}\left(Y;\frac{l}{\alpha }\right)$.*
*2*. *$DRS\Lambda_{\tau_{1},\tau_{2}}\left(Y+\beta ;l\right)=DRS\Lambda_{\tau_{1},\tau_{2}}\left(Y;l-\beta \right)$.*

Let $DRS\Lambda_{\tau_{1},\tau_{2}}\left(Y_{1};l\right)$ and $DRS\Lambda_{\tau_{1},\tau_{2}}\left(Y_{2};l\right)$ represent the dynamic residual cumulative Sharma–Taneja–Mittal entropy of the random variables $Y_{1}$ and $Y_{2}$, where $Y_{1}=\psi \left(Y_{2}\right)$. We now confirm the conditions under which the transformations raise or lower the cumulative Sharma–Taneja–Mittal entropy of the dynamic residual. We specify the dispersion ordered of $Y_{1}$ and $Y_{2}$ to demonstrate this.

**Definition** **1.** A random variable $Y_{1}$ is said to possess a higher degree of dispersion than $Y_{2}$, denoted by $Y_{1}{⪰}^{ODIS}Y_{2}$, if and only if there exists a dilation function ψ(·) such that $Y_{1}=\psi \left(Y_{2}\right)$. This means that the function ψ satisfies $\psi \left(y\right)-\psi \left(y^{*}\right)\geq y-y^{*},\;\mathrm{for}\;\mathrm{all}\;y\geq y^{*}$.

**Theorem** **4.** *If it holds that $Y_{1}{⪰}^{ODIS}Y_{2}$ (or equivalently, $Y_{1}{⪯}^{ODIS}Y_{2}$), then the relationship $DRS\Lambda_{\tau_{1},\tau_{2}}\left(Y_{1};l\right)\leq \left(\geq \right)DRS\Lambda_{\tau_{1},\tau_{2}}\left(Y_{2};l\right)$ is valid.*

**Proof.**  Since the dilation function ψ satisfies $\psi^{\prime }\left(y\right)\geq 1$ for all *y*, and using the non-positivity property established earlier, the required inequality follows immediately from the application of Equation (13). Hence, the result is proved. □

**Theorem** **5.** *Let Y be a random variable that is continuous with hazard rate function $Hr\left(l\right)=\frac{k(l)}{\bar{K}\left(l\right)}$ and the survival function $\bar{K}\left(y\right)$. The connection between the dynamic residual cumulative Sharma–Taneja–Mittal entropy and hazard rate function is provided by*

(14) $$\begin{array}{c} Hr\left(l\right)=\frac{DRS\Lambda_{\tau_{1},\tau_{2}}^{\prime }\left(Y;l\right)}{\tau_{i}\;DRS\Lambda_{\tau_{1},\tau_{2}}\left(Y;l\right)+\int_{l}^{\infty }{\left(\frac{\bar{K}\left(y\right)}{\bar{K}\left(l\right)}\right)}^{\tau_{j}}\;dy}, \end{array}$$

*where i≠j=1,2.*

**Proof.**  From (12), we have

$$\begin{array}{cc} \left(\tau_{1}-\tau_{2}\right)DRS\Lambda_{\tau_{1},\tau_{2}}\left(Y;l\right) & =\int_{l}^{\infty }{\left(\frac{\bar{K}\left(y\right)}{\bar{K}\left(l\right)}\right)}^{\tau_{1}}\;dy-\int_{l}^{\infty }{\left(\frac{\bar{K}\left(y\right)}{\bar{K}\left(l\right)}\right)}^{\tau_{1}}\;dy. \end{array}$$

When we differentiate both sides of the equation above with regard to *l*, we obtain

$$\begin{array}{cc} \left(\tau_{1}-\tau_{2}\right)DRS\Lambda_{\tau_{1},\tau_{2}}^{\prime }\left(Y;l\right) & =-1+\tau_{1}Hr\left(l\right)\int_{l}^{\infty }{\left(\frac{\bar{K}\left(y\right)}{\bar{K}\left(l\right)}\right)}^{\tau_{1}}\;dy+1-\tau_{2}Hr\left(l\right)\int_{l}^{\infty }{\left(\frac{\bar{K}\left(y\right)}{\bar{K}\left(l\right)}\right)}^{\tau_{2}}\;dy, \end{array}$$

which is equivalent to

$$\begin{array}{cc} \left(\tau_{1}-\tau_{2}\right)DRS\Lambda_{\tau_{1},\tau_{2}}^{\prime }\left(Y;l\right) & =Hr\left(l\right)\left[\tau_{1}\left(\tau_{1}-\tau_{2}\right)DRS\Lambda_{\tau_{1},\tau_{2}}\left(Y;l\right)+\left(\tau_{1}-\tau_{2}\right)\int_{l}^{\infty }{\left(\frac{\bar{K}\left(y\right)}{\bar{K}\left(l\right)}\right)}^{\tau_{2}}\;dy\right]. \end{array}$$

Then,

$$\begin{array}{c} Hr\left(l\right)=\frac{DRS\Lambda_{\tau_{1},\tau_{2}}^{\prime }\left(Y;l\right)}{\tau_{1}\;DRS\Lambda_{\tau_{1},\tau_{2}}\left(Y;l\right)+\int_{l}^{\infty }{\left(\frac{\bar{K}\left(y\right)}{\bar{K}\left(l\right)}\right)}^{\tau_{2}}\;dy}, \end{array}$$

which proves the theorem. □

**Proposition** **2.** *Assume that Y is a non-negative variable that is random with a hazardous rate function Hr(y) and the survival function $\bar{K}\left(y\right)$. The survival function $\bar{K}\left(y\right)$ is therefore uniquely determined by $DRS\Lambda_{\tau_{1},\tau_{2}}\left(Y;l\right)$.*

In the following theorem, we demonstrate that the Sharma–Taneja–Mittal entropy’s dynamic residual cumulative can possibly be represented as an expectation of the mean residual function MHr(l).

**Theorem** **6.** *The dynamic residual cumulative Sharma–Taneja–Mittal entropy can be expressed as*

(15) $$\begin{array}{cc} DRS\Lambda_{\tau_{1},\tau_{2}}\left(Y;l\right) & =\frac{1}{\tau_{1}-\tau_{2}}\left[\frac{\left(\tau_{2}-1\right)}{{\bar{K}}^{\tau_{2}}\left(l\right)}E\left(MHr\left(Y\right){\bar{K}}^{\tau_{2}-1}\left(Y\right)|Y>l\right)\right.\\ & \;\left.-\frac{\left(\tau_{1}-1\right)}{{\bar{K}}^{\tau_{1}}\left(l\right)}E\left(MHr\left(Y\right){\bar{K}}^{\tau_{1}-1}\left(Y\right)|Y>l\right)\right]. \end{array}$$

**Proof.**  We can write (12) as

$$\begin{array}{cc} DRS\Lambda_{\tau_{1},\tau_{2}}\left(Y;l\right) & =\frac{1}{\tau_{1}-\tau_{2}}\left[\frac{\left(\tau_{2}-1\right)}{\left(\tau_{2}-1\right)}\left(MHr\left(l\right)-\int_{l}^{\infty }{\left(\frac{\bar{K}\left(y\right)}{\bar{K}\left(l\right)}\right)}^{\tau_{2}}\;dy\right)\right.\\ & \;\left.-\frac{\left(\tau_{1}-1\right)}{\left(\tau_{1}-1\right)}\left(MHr\left(l\right)-\int_{l}^{\infty }{\left(\frac{\bar{K}\left(y\right)}{\bar{K}\left(l\right)}\right)}^{\tau_{1}}\;dy\right)\right]. \end{array}$$

By noting that

$$\begin{array}{cc} \int_{l}^{\infty }{\bar{K}}^{\tau_{i}}\left(y\right)\;dy & =\int_{l}^{\infty }\left(\frac{d}{dy}\left(MHr\left(y\right)\;\bar{K}\left(y\right)\right)\right){\bar{K}}^{\tau_{i}-1}\left(y\right)\;dy\\ & =-MHr\left(l\right){\bar{K}}^{\tau_{i}}\left(l\right)+\left(\tau_{i}-1\right)\int_{l}^{\infty }MHr\left(y\right){\bar{K}}^{\tau_{i}-1}\left(y\right)k\left(y\right)\;dy, \end{array}$$

where i=1,2, then the result follows. □

The equilibrium random and the initial random variables are related by the following theorem.

**Theorem** **7.** *Assume that the cdf K has the decreasing mean residual life property. Then the equilibrium random variable $Y_{Eb}$ obeys the inequality*

(16) $$\left(\tau_{1}-\tau_{2}\right)DRS\Lambda_{\tau_{1},\tau_{2}}\left(Y_{Eb};l\right)\geq DRS\Lambda_{\tau_{1},\tau_{2}}\left(Y;l\right),\;\tau_{1}>\tau_{2}>1.$$

**Proof.**  The survival function for the equilibrium’s randomly selected variable $Y_{Eb}$ is provided by

$${\bar{K}}_{Y_{Eb}}\left(l\right)=\frac{MHr\left(l\right)\bar{K}\left(l\right)}{\mu },\mathrm{where}\;\mu =E\left(Y\right).$$

$$\begin{array}{cc} \left(\tau_{1}-\tau_{2}\right)DRS\Lambda_{\tau_{1},\tau_{2}}\left(Y_{Eb};l\right) & =\int_{l}^{\infty }{\left[{\bar{K}}_{Y_{Eb}}\left(y\right)-{\bar{K}}_{Y_{Eb}}\left(y\right)\right]}^{\tau_{1}}\;dy\\ \; & =\frac{1}{{\left(MHr\left(l\right)\bar{K}\left(l\right)\right)}^{\tau_{1}}}\int_{l}^{\infty }{\left(MHr\left(y\right)\bar{K}\left(y\right)\right)}^{\tau_{1}}\;dy\\ \; & \;-\frac{1}{{\left(MHr\left(l\right)\bar{K}\left(l\right)\right)}^{\tau_{2}}}\int_{l}^{\infty }{\left(MHr\left(y\right)\bar{K}\left(y\right)\right)}^{\tau_{2}}\;dy. \end{array}$$

Moreover, for the non-positivity property, the equation above now simplifies to (16) when the decreasing mean residual life property is applied. □

## 4. Alternative Dynamic Residual Cumulative Sharma–Taneja–Mittal Entropy

In this section, we will discuss an alternative version of the dynamic residual cumulative Sharma–Taneja–Mittal entropy. In analogy with (4), we can define the alternative dynamic residual cumulative Sharma–Taneja–Mittal entropy as follows:

(17) $$\begin{array}{cc} DRS\Lambda_{\tau_{1},\tau_{2}}^{*}\left(Y;l\right) & =\frac{1}{\tau_{1}-\tau_{2}}\left[\int_{0}^{\infty }k_{l}^{\tau_{1}}\left(y\right)\;dy-\int_{0}^{\infty }k_{l}^{\tau_{1}}\left(y\right)\;dy\right]\\ & =\frac{1}{\tau_{1}-\tau_{2}}\left[\int_{l}^{\infty }{\left(\frac{k(y)}{\bar{K}\left(l\right)}\right)}^{\tau_{1}}\;dy-\int_{l}^{\infty }{\left(\frac{k(y)}{\bar{K}\left(l\right)}\right)}^{\tau_{2}}\;dy\right]\\ & =\frac{1}{\tau_{1}-\tau_{2}}\left[\int_{0}^{1}k_{l}^{\tau_{1}-1}\left({\bar{K}}_{l}^{-1}\left(u\right)\right)\;du-\int_{0}^{1}k_{l}^{\tau_{2}-1}\left({\bar{K}}_{l}^{-1}\left(u\right)\right)\;du\right], \end{array}$$

with the condition that $\tau_{1}\neq \tau_{2}>0$, and ${\bar{K}}_{l}^{-1}\left(u\right)=\inf\left\{y;{\bar{K}}_{l}\left(y\right)\geq u\right\}$ is the quantile function of ${\bar{K}}_{l}\left(y\right)=\frac{\bar{K}\left(y+l\right)}{\bar{K}\left(l\right)}$, y,l>0. We readily recognize that $DRS\Lambda_{\tau_{1},\tau_{2}}^{*}\left(Y;0\right)=S\Lambda_{\tau_{1},\tau_{2}}\left(Y\right)$ at l=0.

The resultant value of the expression $DRS\Lambda_{\tau_{1},\tau_{2}}^{*}\left(.;l\right)$ under affine translation is provided by the following lemma. The proof is left out.

**Lemma** **2.** *Define Z=αY+β for every absolutely continuous random variable Y, where α>0 and β≥0 are the constants. Subsequently,*

$$DRS\Lambda_{\tau_{1},\tau_{2}}^{*}\left(Z;l\right)=\frac{1}{\tau_{1}-\tau_{2}}\left[\frac{1}{\alpha^{\tau_{1}-1}}\int_{\frac{l-\beta }{\alpha }}^{\infty }{\left(\frac{k(y)}{\bar{K}\left(\frac{l-\beta }{\alpha }\right)}\right)}^{\tau_{1}}\;dy-\frac{1}{\alpha^{\tau_{2}-1}}\int_{\frac{l-\beta }{\alpha }}^{\infty }{\left(\frac{k(y)}{\bar{K}\left(\frac{l-\beta }{\alpha }\right)}\right)}^{\tau_{2}}\;dy\right].$$

**Proof.**  Using the transformation for the pdf, $k\left(\alpha y+\beta \right)=\frac{1}{\alpha }k\left(y\right)$, so

$${\left(\frac{k(\alpha y+\beta )}{\bar{K}\left(l\right)}\right)}^{\tau_{i}}={\left(\frac{\frac{1}{\alpha }k\left(y\right)}{\bar{K}\left(l\right)}\right)}^{\tau_{i}}=\frac{1}{\alpha^{\tau_{i}}}{\left(\frac{k(y)}{\bar{K}\left(l\right)}\right)}^{\tau_{i}},\;i=1,2.$$

Then, the result follows. □

Ebrahimi [6] introduced two non-parametric distribution classes characterized by the function $DR\Lambda^{*}\left(Y;l\right)$.

**Definition** **2.** A random variable Y is said to exhibit a decreasing (or increasing) life residual property—denoted as DLR (or ILR)—if the function $DR\Lambda^{*}\left(Y;l\right)$ is monotonically decreasing (or increasing) for every l≥0.

Similarly, using the measure $DRS\Lambda_{\tau_{1},\tau_{2}}^{*}\left(Y;l\right)$, we define another set of nonparametric classes.

**Definition** **3.** A non-negative random variable Y is classified as having a decreasing (or increasing) life residual—referred to as DLR (or ILR)—if the function $DRS\Lambda_{\tau_{1},\tau_{2}}^{*}\left(Y;l\right)$ decreases (or increases) continuously for all l≥0.

We give a counter-example below to demonstrate that not every distribution is monotonous with regard to $DR\Lambda^{*}\left(Y;l\right)$.

**Example** **3.** *(Illustrative Counterexample) Consider a nonnegative random variable Y whose survival function is defined as*

$$\bar{K}\left(y\right)=1-\left[\left(1-e^{-y}\right)\left(1-e^{-2y}\right)\right],\;y\geq 0.$$

*Accordingly, the probability density function of Y is given by*

$$k\left(y\right)=e^{-y}+2e^{-2y}-3e^{-3y},\;y>0.$$

**Figure 4:**
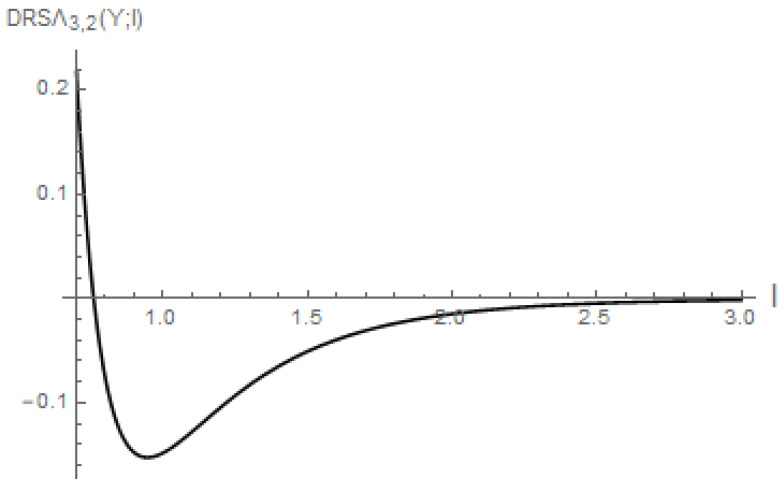
Plot of $DRS\Lambda_{\tau_{1},\tau_{2}}^{*}\left(Y;l\right)$ (Example 3) with $\tau_{1}=3$ and $\tau_{2}=2$ against l∈(0,3).

*Now, set $\tau_{1}=3$ and $\tau_{2}=2$. Then, the expression*

$$\begin{array}{cc} DRS\Lambda_{\tau_{1},\tau_{2}}^{*}\left(Y;l\right) & =\frac{1}{\tau_{1}-\tau_{2}}\left[\int_{l}^{\infty }{\left(\frac{k(y)}{\bar{K}\left(l\right)}\right)}^{\tau_{1}}\;dy-\int_{l}^{\infty }{\left(\frac{k(y)}{\bar{K}\left(l\right)}\right)}^{\tau_{2}}\;dy\right]\\ \; & =\frac{1}{\tau_{1}-\tau_{2}}\left[\int_{l}^{\infty }{\left(\frac{e^{-y}+2e^{-2y}-3e^{-3y}}{e^{-l}+e^{-2l}-e^{-3l}}\right)}^{\tau_{1}}dy\right.\\ \; & \;\;\left.-\int_{l}^{\infty }{\left(\frac{e^{-y}+2e^{-2y}-3e^{-3y}}{e^{-l}+e^{-2l}-e^{-3l}}\right)}^{\tau_{2}}dy\right]. \end{array}$$

is obtained. As illustrated in Figure 4, this function $DRS\Lambda_{\tau_{1},\tau_{2}}^{*}\left(Y;l\right)$ is not monotonic.

**Remark** **1.** Unlike Proposition 1, we find that $DRS\Lambda_{\tau_{1},\tau_{2}}^{*}\left(Y;l\right)$ is not always non-positive, as shown in Figure 4. This can be explained by the fact that the ratio $\frac{k(y)}{\bar{K}\left(l\right)}$ is not always less than or equal to one (not necessarily bounded above by one).

### 4.1. Record Values

Let us look at a technical mechanism that experiences shocks like spikes in voltage. Given a common continual cdf K(.), pdf k(.), and survival function $\bar{K}\left(l\right)=1-K\left(l\right)$, the shocks may then be treated as a series of independently and equally distributed random variables $\left\{Y_{j},j\geq 1\right\}$. The stresses placed on the mechanism at various points in time are represented by the shocks. The record statistics of this sequence, or the values of the greatest stresses recorded thus far, are of interest to us. Let us represent the *j*-th ordering statistic from the first *p* occurrences by $Y_{j:p}$. Next, we establish the sequences of upper record values $R_{\left(p\right)}$ and upper record timings $L_{p},\left(p\geq 1\right)$, as follows:

$$R_{\left(p\right)}=Y_{L_{p}:L_{p}},\;p=0,1,\ldots ,$$

noting that

$$L_{0}=1,\;L_{p}=\min\left\{i:i>L_{p-1},Y_{i}>R_{p}\right\},\;p\geq 1.$$

Knowing the incomplete gamma function,

$$I\Gamma \left(\alpha ,y\right)=\int_{y}^{\infty }v^{\alpha -1}e^{-v}\;dv,\;\alpha ,y>0,$$

we can see that the survival function and the pdf of $R_{\left(p\right)}$, represented by $k_{R_{\left(p\right)}}\left(y\right)$ and ${\bar{K}}_{R_{\left(p\right)}}\left(y\right)$, correspondingly, are known to be provided by (see, e.g., [22])

(18) $$k_{R_{\left(p\right)}}\left(y\right)=\frac{{\left[-\ln\bar{K}\left(y\right)\right]}^{p-1}}{(p-1)!}k\left(y\right),\;y\geq 0,$$

and

(19) $${\bar{K}}_{R_{\left(p\right)}}\left(y\right)=\bar{K}\left(y\right)\sum_{t=0}^{p-1}\frac{{\left[-\log\bar{K}\left(y\right)\right]}^{t}}{t!}=\frac{I\Gamma (p,-\log\bar{K}\left(y\right))}{(p-1)!},\;y\geq 0.$$

In this part, our focus is on analyzing an alternative formulation of the dynamic residual cumulative Sharma–Taneja–Mittal entropy associated with the random variable $Y_{R_{\left(p\right)}}$. This measure serves to assess the level of uncertainty arising from the density function of $\left[Y_{R_{\left(p\right)}}-l|Y_{R_{\left(p\right)}}>l\right]$, which characterizes the remaining lifetime of the system and its predictability. To simplify the computational process, we present a lemma establishing the connection between this alternative formulation of dynamic residual cumulative Sharma–Taneja–Mittal entropy for record value from a uniform distribution and the incomplete gamma function. This link is practically significant as it facilitates a more efficient computation of the proposed entropy measure.

**Lemma** **3.** *Consider a sequence $\left\{R_{j},j\geq 1\right\}$ of independent and identically distributed random variables following a uniform distribution. Define ${\overset{ˇ}{R}}_{\left(p\right)}$ as the p-th upper record value within this sequence. Then, the following holds:*

(20) $$\begin{array}{cc} DRS\Lambda_{\tau_{1},\tau_{2}}^{*}\left({\overset{ˇ}{R}}_{\left(p\right)};l\right) & =\frac{1}{\tau_{1}-\tau_{2}}\left[\frac{I\Gamma (\tau_{1}\left(p-1\right)+1,-\ln\left(1-l\right))}{I\Gamma^{\tau_{1}}\left(p,-\ln\left(1-l\right)\right)}-\frac{I\Gamma (\tau_{2}\left(p-1\right)+1,-\ln\left(1-l\right))}{I\Gamma^{\tau_{2}}\left(p,-\ln\left(1-l\right)\right)}\right], \end{array}$$

*for all $\tau_{1}\neq \tau_{2}>0$ and 0<l<1.*

**Proof.**  For the given sequence $\left\{R_{j},j\geq 1\right\}$, the random variables are uniformly distributed on (0,1), so the survival function is

$$\bar{K}\left(y\right)=1-y,\;0<y<1.$$

Using (18) and (19), we have

$$\begin{array}{cc} I^{*}=\int_{l}^{1}{\left[\frac{k_{{\overset{ˇ}{R}}_{\left(p\right)}}\left(y\right)}{{\bar{K}}_{{\overset{ˇ}{R}}_{\left(p\right)}}\left(l\right)}\right]}^{\tau_{i}}\;dy & =\int_{l}^{1}{\left(\frac{{\left[-\ln\left(1-y\right)\right]}^{p-1}}{I\Gamma (p,-\ln(1-l))}\right)}^{\tau_{i}}dy,\;i=1,2. \end{array}$$

Changing the variable,

$$v=-\ln\left(1-y\right),\;dv=\frac{dy}{1-y}=e^{-v}dv.$$

Rewriting the integral in terms of *v*, where $y=1-e^{-v}$, so when y=l, we obtain v=−ln(1−l):

$$\begin{array}{cc} I^{*} & =\int_{-\ln(1-l)}^{1}{\left(\frac{v^{p-1}}{I\Gamma (p,-\ln(1-l))}\right)}^{\tau_{i}}e^{-v}dv\\ & =\int_{-\ln(1-l)}^{1}\frac{v^{\tau_{i}\left(p-1\right)}e^{-v}}{I\Gamma^{\tau_{i}}\left(p,-\ln\left(1-l\right)\right)}dv. \end{array}$$

Recognizing this integral as an incomplete gamma function,

$$I^{*}=\frac{I\Gamma (\tau_{i}\left(p-1\right)+1,-\ln\left(1-l\right))}{I\Gamma^{\tau_{i}}\left(p,-\ln\left(1-l\right)\right)}.$$

□

**Figure 5:**
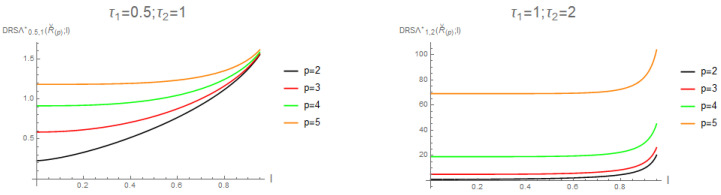
For $\tau_{1}=0.5;\tau_{2}=1$ (**left**) and $\tau_{1}=1;\tau_{2}=2$ (**right**), the precise values of $DRS\Lambda_{\tau_{1},\tau_{2}}^{*}\left({\overset{ˇ}{R}}_{\left(p\right)};l\right)$ with regard to 0<l<1.

Practitioners and researchers may easily use the well-known incomplete gamma function to calculate the alternative formulation of the dynamic residual cumulative Sharma–Taneja–Mittal entropy of recording values from a distribution that is uniform by utilizing this lemma. The measure’s applicability and usability in a variety of scenarios are improved by this computational reduction. For the amounts of $\tau_{1}=0.5;\tau_{2}=1$ and $\tau_{1}=1;\tau_{2}=2$ together with the value of p=2,…,5, we show the plot of $DRS\Lambda_{\tau_{1},\tau_{2}}^{*}\left({\overset{ˇ}{R}}_{\left(p\right)};l\right)$ in Figure 5.

We denote $V∼T\Gamma_{l}\left(\alpha ,\beta \right)$ to signify that the random variable *V* follows a truncated Gamma distribution, characterized by the pdf

$$k_{V}\left(v\right)=\frac{\beta^{\alpha }}{I\Gamma (\alpha ,l)}v^{\alpha -1}e^{-\beta v},\;v>l>0,$$

where α and β are both positive parameters. The following theorem establishes a connection between the alternative expression of the dynamic residual cumulative Sharma–Taneja–Mittal entropy for record values $R_{\left(p\right)}$ and this entropy measure for record values derived from a distribution of uniform.

**Theorem** **8.** *A series of independently and equally distributed variables that are random, having cdf K and pdf k, is represented as $\left\{Y_{j},j\geq 1\right\}$. For the sequence $\left\{Y_{j}\right\}$, let $R_{\left(p\right)}$ represent the p-th higher record value. Then, for any $\tau_{1}\neq \tau_{2}>0$, the alternative expression of the dynamic residual cumulative Sharma–Taneja–Mittal entropy of $R_{\left(p\right)}$ is constructed as follows:*

(21) $$\begin{array}{cc} DRS\Lambda_{\tau_{1},\tau_{2}}^{*}\left(R_{\left(p\right)};l\right) & =\frac{1}{\tau_{1}-\tau_{2}}\left[\left(\tau_{1}-\tau_{2}\right)DRS\Lambda_{\tau_{1},\tau_{2}}^{*}\left({\overset{ˇ}{R}}_{\left(p\right)};K\left(l\right)\right)E\left[k^{\tau_{1}-1}\left(K^{-1}\left(1-e^{-V_{\left(p\right)}^{\left(1\right)}}\right)\right)\right]\right.\\ & \left.+\frac{I\Gamma (\tau_{2}\left(p-1\right)+1,-\ln\left(1-l\right))}{I\Gamma^{\tau_{2}}\left(p,-\ln\left(1-l\right)\right)}\left(E[k^{\tau_{1}-1}\left(K^{-1}\left(1-e^{-V_{\left(p\right)}^{\left(1\right)}}\right)\right)]\right.\right.\\ & \left.\left.-E[k^{\tau_{2}-1}\left(K^{-1}\left(1-e^{-V_{\left(p\right)}^{\left(2\right)}}\right)\right)]\right)\right],\;l>0, \end{array}$$

*where $V_{\left(p\right)}^{\left(i\right)}∼T\Gamma_{-\ln\bar{K}\left(l\right)}\left(\tau_{i}\left(p-1\right)+1,1\right)$, i=1,2.*

**Proof.**  Using (18) and (19), we have

$$\begin{array}{cc} \int_{l}^{\infty }{\left[\frac{k_{R_{\left(p\right)}}\left(y\right)}{{\bar{K}}_{R_{\left(p\right)}}\left(l\right)}\right]}^{\tau_{i}}\;dy & =\int_{l}^{\infty }{\left(\frac{{\left[-\ln\left(1-K\left(y\right)\right)\right]}^{p-1}k\left(y\right)}{I\Gamma (p,-\ln(1-K(l)))}\right)}^{\tau_{i}}dy\\ & =\frac{I\Gamma (\tau_{i}\left(p-1\right)+1,-\ln\left(1-K\left(l\right)\right))}{I\Gamma^{\tau_{i}}\left(p,-\ln\left(1-K\left(l\right)\right)\right)}\int_{K(l)}^{1}\\ & \;\;\;\times \frac{{\left(-\ln\left(1-u\right)\right)}^{\tau_{i}\left(p-1\right)}k^{\tau_{i}-1}\left(K^{-1}\left(u\right)\right)}{I\Gamma (\tau_{i}\left(p-1\right)+1,-\ln\left(1-K\left(l\right)\right))}\;du\\ & =\frac{I\Gamma (\tau_{i}\left(p-1\right)+1,-\ln\left(1-K\left(l\right)\right))}{I\Gamma^{\tau_{i}}\left(p,-\ln\left(1-K\left(l\right)\right)\right)}\\ & \;\;\;\times \int_{-\ln(1-K(l))}^{\infty }\frac{z^{\tau_{i}\left(p-1\right)}e^{-z}k^{\tau_{i}-1}\left(K^{-1}\left(1-e^{-z}\right)\right)}{I\Gamma (\tau_{i}\left(p-1\right)+1,-\ln\left(1-K\left(l\right)\right))}\;dz, \end{array}$$

where i=1,2. Then, by applying Lemma 3, the proof is completed. □

The following example shows the application of the previous theorem.

**Example** **4.** *We assume a series of randomly distributed variables with equal and independent distributions $\left\{Y_{j},j\geq 1\right\}$ that have a similar Weibull distribution. This distribution’s cdf is provided by*

$$K\left(y\right)=1-e^{-y^{3}},\;y>0.$$

*The inverse cdf of Y may be found as*

$$K^{-1}\left(v\right)={\left(-\ln\left(1-v\right)\right)}^{1/3},\;0<v<1.$$

*After that, we may compute*

$$E\left[k^{\tau_{i}-1}\left(K^{-1}\left(1-e^{-V_{\left(p\right)}^{\left(i\right)}}\right)\right)\right]=\frac{3^{\tau_{i}-1}}{\tau_{i}^{\tau_{i}\left(p-\frac{1}{3}\right)+\frac{1}{3}}}\frac{I\Gamma (\tau_{i}\left(p-\frac{1}{3}\right)+\frac{1}{3},\tau_{i}l^{3})}{I\Gamma (\tau_{i}\left(p-1\right)+1,l^{3})},\;i=1,2.$$

*Thus, utilizing (21), we obtain*

$$DRS\Lambda_{\tau_{1},\tau_{2}}^{*}\left(R_{\left(p\right)};l\right)=\frac{1}{\tau_{1}-\tau_{2}}\left[\frac{3^{\tau_{1}-1}I\Gamma \left(\tau_{1}\left(p-\frac{1}{3}\right)+\frac{1}{3},\tau_{1}l^{3}\right)}{\tau_{1}^{\tau_{1}\left(p-\frac{1}{3}\right)+\frac{1}{3}}I\Gamma^{\tau_{1}}\left(p,l^{3}\right)}-\frac{3^{\tau_{2}-1}I\Gamma \left(\tau_{2}\left(p-\frac{1}{3}\right)+\frac{1}{3},\tau_{2}l^{3}\right)}{\tau_{2}^{\tau_{2}\left(p-\frac{1}{3}\right)+\frac{1}{3}}I\Gamma^{\tau_{2}}\left(p,l^{3}\right)}\right],$$

*p≥1.*

### 4.2. Conditional Entropy of Record Values

Given that all units have voltages greater than l>0, we are now interested in assessing the remaining recordings $Y_{R_{\left(p\right)}}-l$, l≥0. Thus, $Y_{R_{\left(p\right)},l}^{0}=\left[Y_{R_{\left(p\right)}}-l|Y_{R_{\left(0\right)}}>l\right]$ may express its survival function as follows, see [23],

$$K_{R_{\left(p\right)},l}\left(y\right)=P\left(Y_{R_{\left(p\right)}}-l>y|Y_{R_{\left(0\right)}}>l\right)=I\Gamma \left(p+1,-\ln{\bar{K}}_{l}\left(y\right)\right).$$

Consequently, we possess

(22) $$k_{R_{\left(p\right)},l}\left(y\right)=\frac{{\left[-\ln{\bar{K}}_{l}\left(y\right)\right]}^{p-1}}{(p-1)!}k_{t}\left(x\right),\;y\geq l\geq 0.$$

In the following part, we will examine the Sharma–Taneja–Mittal entropy of the random variable $Y_{R_{\left(p\right)},l}^{0}$, which quantifies the degree of uncertainty regarding the predicted duration of the framework’s residual lifetime with regard to the Sharma–Taneja–Mittal entropy contained in the density of $\left[Y_{R_{\left(p\right)}}-l|Y_{R_{\left(0\right)}}>l\right]$. A key component of our goal is the probability of the integral substitution $V={\bar{K}}_{l}\left(Y_{R_{\left(p\right)},l}^{0}\right)$. ${\overset{ˇ}{R}}_{\left(p\right)}={\bar{K}}_{l}\left(Y_{R_{\left(p\right)},l}^{0}\right)$ obviously has the following pdf:

(23) $$h_{R_{\left(p\right)}}\left(v\right)=\frac{{\left(-\ln\left(1-v\right)\right)}^{p-1}}{(p-1)!},\;0<v<1,\;p\geq 1.$$

In the next proposal, we use the previously described transformations to give an expression for the alternative formulation of the dynamic residual cumulative Sharma–Taneja–Mittal entropy of $Y_{R_{\left(p\right)},l}^{0}$.

**Theorem** **9.** *A series of independently and equally distributed variables that are random, having cdf K and pdf k, is represented as $\left\{Y_{j},j\geq 1\right\}$. The alternative expression of the dynamic residual cumulative Sharma–Taneja–Mittal entropy of $Y_{R_{\left(p\right)},l}^{0}$ is able to be stated as follows:*

$$DRS\Lambda_{\tau_{1},\tau_{2}}^{*}\left(Y_{R_{\left(p\right)},l}^{0}\right)=\frac{1}{\tau_{1}-\tau_{2}}\left[\int_{0}^{1}h_{R_{\left(p\right)}}^{\tau_{1}}\left(v\right)k_{l}^{\tau_{1}-1}\left({\bar{K}}_{l}^{-1}\left(v\right)\right)\;dv-\int_{0}^{1}h_{R_{\left(p\right)}}^{\tau_{2}}\left(v\right)k_{l}^{\tau_{2}-1}\left({\bar{K}}_{l}^{-1}\left(v\right)\right)\;dv\right],$$

*for every $\tau_{1}\neq \tau_{2}>0$, l>0.*

**Proof.**  From (17) and (22), we use the changed value of the variable $v={\bar{K}}_{l}\left(y\right)$ to obtain

$$\begin{array}{cc} \left(\tau_{1}-\tau_{2}\right)DRS\Lambda_{\tau_{1},\tau_{2}}^{*}\left(Y_{R_{\left(p\right)},l}^{0}\right) & =\int_{0}^{\infty }{\left(k_{Y_{R_{\left(p\right)},l}^{0}}\right)}^{\tau_{1}}\;dy-\int_{0}^{\infty }{\left(k_{Y_{R_{\left(p\right)},l}^{0}}\right)}^{\tau_{2}}\;dy\\ & =\int_{0}^{\infty }{\left(\frac{{\left[-\ln{\bar{K}}_{l}\left(y\right)\right]}^{p-1}}{(p-1)!}k_{(}y\left|l\right)\right)}^{\tau_{1}}\;dy\\ & \;\;\;-\int_{0}^{\infty }{\left(\frac{{\left[-\ln{\bar{K}}_{l}\left(y\right)\right]}^{p-1}}{(p-1)!}k_{(}y\left|l\right)\right)}^{\tau_{2}}\;dy\\ & =\int_{0}^{1}{\left(\frac{{\left[-\ln\left(1-v\right)\right]}^{p-1}}{(p-1)!}\right)}^{\tau_{1}}k_{l}^{\tau_{1}-1}\left({\bar{K}}_{l}^{-1}\left(v\right)\right)\;dv\\ & \;\;\;-\int_{0}^{1}{\left(\frac{{\left[-\ln\left(1-v\right)\right]}^{p-1}}{(p-1)!}\right)}^{\tau_{2}}k_{l}^{\tau_{2}-1}\left({\bar{K}}_{l}^{-1}\left(v\right)\right)\;dv\\ & =\int_{0}^{1}h_{R_{\left(p\right)}}^{\tau_{1}}\left(v\right)k_{l}^{\tau_{1}-1}\left({\bar{K}}_{l}^{-1}\left(v\right)\right)\;dv-\int_{0}^{1}h_{R_{\left(p\right)}}^{\tau_{2}}\left(v\right)k_{l}^{\tau_{2}-1}\left({\bar{K}}_{l}^{-1}\left(v\right)\right)\;dv. \end{array}$$

The final equality completes the proof since $h_{R_{\left(p\right)}}\left(v\right)$ represents the pdf of *V* stated in (23). □

## 5. Entropy of Sharma–Taneja–Mittal of a Combination of Systems That Are Coherent

Based on the definition of continuous Sharma–Taneja–Mittal entropy given in Equation (5), we can express the continuous Sharma–Taneja–Mittal entropy of a random variable *Y* as follows:

**Proposition** **3.** *Consider Y as a continuous random variable characterized by a probability density function (pdf) k(y). Then, an alternative formulation of Sharma–Taneja–Mittal entropy using the hazard rate function, defined as $Hr\left(l\right)=\frac{k(l)}{\bar{K}\left(l\right)}$, is presented below:*

(24) $$\begin{array}{cc} S\Lambda \tau_{1},\tau_{2}\left(Y\right) & =\frac{1}{\tau_{1}-\tau_{2}}\int_{0}^{\infty }\left(k^{\tau_{1}}\left(y\right)-k^{\tau_{2}}\left(y\right)\right)dy\\ & =\frac{1}{\tau_{1}-\tau_{2}}\left(E\left[{\left(Hr\left(Y_{\tau_{1}}\right)\right)}^{\tau_{1}-1}\right]-E\left[{\left(Hr\left(Y_{\tau_{2}}\right)\right)}^{\tau_{2}-1}\right]\right), \end{array}$$

*where the pdf of the transformed random variable $Y_{\tau_{i}}$, for i=1,2, is given by*

$$k_{Y_{\tau_{i}}}\left(y\right)=\tau_{i}{\bar{K}}^{\tau_{i}-1}\left(y\right)k\left(y\right).$$

Following the approach of Shaked and Shanthikumar [24], with the addition of Definition 1, we make use of specific stochastic order relations, including the stochastic order (${⪰}^{OST}$), the hazard rate order (${⪰}^{OHR}$), and the dispersive order (${⪰}^{ODIS}$). Furthermore, the relationships among these orders can be summarized as

1. ${⪰}^{OHR}⟹{⪰}^{OST}$;
2. ${⪰}^{ODIS}⟹{⪰}^{OST}$.

**Definition** **4.** *Let $Y_{1}$ and $Y_{2}$ be two non-negative continuous random variables with pdfs $k_{1}\left(y\right)$ and $k_{2}\left(y\right)$, respectively. Then, we say that $Y_{1}$ is smaller than $Y_{2}$ in the sense of Sharma–Taneja–Mittal entropy, denoted as $Y_{1}{⪰}^{STM}Y_{2}$, if*

$$S\Lambda \tau_{1},\tau_{2}\left(Y_{1}\right)\leq S\Lambda \tau_{1},\tau_{2}\left(Y_{2}\right),$$

*where $\tau_{1}\neq \tau_{2}>0$.*

**Remark** **2.** *The following results will be discussed under the conditions $\tau_{1}-\tau_{2}>1$ and $k{\left(y\right)}^{\tau_{1}}>k{\left(y\right)}^{\tau_{2}}$ for all y>0. Moreover, these conditions hold for any other form of the pdf.*

**Theorem** **10.** *Suppose that $Y_{1}$ and $Y_{2}$ are non-negative continuous random variables, with pdfs $k_{1}$, $k_{2}$ and cdfs $K_{1}$, $K_{2}$, respectively. From (24), if $Y_{1}{⪰}^{ODIS}Y_{2}$, then $Y_{1}{⪰}^{STM}Y_{2}$, noting that the conditions in Remark 2 hold.*

**Proof.**  From (24) with $\tau_{1}-\tau_{2}>1$. If $Y_{1}{⪰}^{ODIS}Y_{2}$, then

$$\begin{array}{cc} \left(\tau_{1}-\tau_{2}\right)\left(S\Lambda \tau_{1},\tau_{2}\left(Y_{2}\right)\right) & =\int_{0}^{1}k_{2}^{\tau_{1}-1}\left(K_{2}^{-1}\left(v\right)\right)dv-\int_{0}^{1}k_{2}^{\tau_{2}-1}\left(K_{2}^{-1}\left(v\right)\right)dv\\ & \leq \int_{0}^{1}k_{1}^{\tau_{1}-1}\left(K_{1}^{-1}\left(v\right)\right)dv-\int_{0}^{1}k_{1}^{\tau_{2}-1}\left(K_{1}^{-1}\left(v\right)\right)dv=\left(\tau_{1}-\tau_{2}\right)\left(S\Lambda \tau_{1},\tau_{2}\left(Y_{1}\right)\right). \end{array}$$

Then, the result follows. □

Aspects of the Sharma–Taneja–Mittal entropy of coherence (and mixing) structures are presented in this section. A system is said to be coherent if its structure–function is monotonic and all of its parts are pertinent. One specific example of a coherence structure is the r-out-of-p system. Additionally, according to Samaniego [25], a collection of coherent frameworks is regarded as a mixed system. In the independent and identical situation, the reliable functional of the mixing system lifespan *L* is reflected by

$${\bar{K}}_{L}\left(l\right)=\sum_{r=1}^{p}\delta_{r}{\bar{K}}_{r:p}\left(l\right),$$

where the survival function of $Y_{1:p},\ldots ,Y_{p:p}$ is K¯r:p(l)=∑i=0r−1piKi(l)K¯p−i(l), r=1,2,…,p. The mixing system lifespan *L*’s pdf is provided by

(25) $$\begin{array}{c} k_{L}\left(l\right)=\sum_{r=1}^{p}\delta_{r}k_{r:p}\left(l\right), \end{array}$$

where $k_{r:p}\left(l\right)$ is defined as, 1≤r≤p,

$$k_{r:p}\left(l\right)=\frac{\Gamma (p+1)}{\Gamma (r)\Gamma (p-r+1)}K^{r-1}\left(l\right){\bar{K}}^{p-r}\left(l\right)k\left(l\right).$$

The vector $\delta =(\delta_{1},...,\delta_{n})$ represents the mechanism’s signature, and $\delta_{r}=P\left(L=Y_{r:p}\right)$, $\sum_{r=1}^{p}\delta_{r}=1$, 1≤r≤n. The pdf of the order of the statistical process $V_{r:p}=K\left(Y_{r:p}\right)$, 1≤r≤p, is

$$h_{r}\left(v\right)=\frac{\Gamma (p+1)}{\Gamma (r)\Gamma (p-r+1)}v^{r-1}{\left(1-v\right)}^{p-r}.$$

Consequently, V=K(L)’s pdf is

(26) $$\begin{array}{c} h_{V}\left(v\right)=\sum_{r=1}^{p}\delta_{r}h_{r}\left(v\right). \end{array}$$

The Sharma–Taneja–Mittal entropy of *L* is discussed in the following formula utilizing the above transformations.

**Theorem** **11.** *The mixed system lifespan L’s Sharma–Taneja–Mittal entropy is*

(27) $$\begin{array}{cc} S\Lambda \tau_{1},\tau_{2}\left(L\right) & =\frac{1}{\tau_{1}-\tau_{2}}\left(\int_{0}^{1}h_{V}^{\tau_{1}}\left(v\right)k^{\tau_{1}-1}\left(K^{-1}\left(v\right)\right)dv-\int_{0}^{1}h_{V}^{\tau_{2}}\left(v\right)k^{\tau_{2}-1}\left(K^{-1}\left(v\right)\right)dv\right), \end{array}$$

*noting that $h_{V}\left(v\right)$ is specified in Equation (26).*

**Proof.**  After applying the transformation v=K(l) to (24), we obtain

(28) $$\begin{array}{cc} S\Lambda \tau_{1},\tau_{2}\left(L\right) & =\frac{1}{\tau_{1}-\tau_{2}}\left(\int_{0}^{\infty }{\left(\sum_{r=1}^{p}\delta_{r}k_{r:p}\left(l\right)\right)}^{\tau_{1}}dl-\int_{0}^{\infty }{\left(\sum_{r=1}^{p}\delta_{r}k_{r:p}\left(l\right)\right)}^{\tau_{2}}dl\right)\\ & =\frac{1}{\tau_{1}-\tau_{2}}\left(\int_{0}^{1}{\left(\sum_{j=1}^{n}\delta_{r}\frac{\Gamma (p+1)}{\Gamma (r)\Gamma (p-r+1)}v^{r-1}{\left(1-v\right)}^{p-r}\right)}^{\tau_{1}}k^{\tau_{1}-1}\left(K^{-1}\left(v\right)\right)dv\right.\\ & \left.-\int_{0}^{1}{\left(\sum_{j=1}^{n}\delta_{r}\frac{\Gamma (p+1)}{\Gamma (r)\Gamma (p-r+1)}v^{r-1}{\left(1-v\right)}^{p-r}\right)}^{\tau_{2}}k^{\tau_{2}-1}\left(K^{-1}\left(v\right)\right)dv\right)\\ & =\frac{1}{\tau_{1}-\tau_{2}}\left(\int_{0}^{1}h_{V}^{\tau_{1}}\left(v\right)k^{\tau_{1}-1}\left(K^{-1}\left(v\right)\right)dv-\int_{0}^{1}h_{V}^{\tau_{2}}\left(v\right)k^{\tau_{2}-1}\left(K^{-1}\left(v\right)\right)dv\right). \end{array}$$

□

**Theorem** **12.** *Assume that two mixed systems with p equally and independent component lives have lifetimes of $L_{Y_{1}}$ and $L_{Y_{2}}$ under the same signature. Next up is the following:*

*1*. *If $Y_{1}{⪰}^{ODIS}Y_{2}$, then $L_{Y_{1}}{⪰}^{STM}L_{Y_{2}}$.*
*2*. *Consider the sets $W_{1}=\left\{0<v<1|\frac{k_{2}\left(K_{2}^{-1}\left(v\right)\right)}{k_{1}\left(K_{1}^{-1}\left(v\right)\right)}<1\right\}$ and $W_{2}=\left\{0<v<1|\frac{k_{2}\left(K_{2}^{-1}\left(v\right)\right)}{k_{1}\left(K_{1}^{-1}\left(v\right)\right)}\geq 1\right\}$. If the relation $Y_{1}{⪰}^{STM}Y_{2}$ holds, then it follows that $L_{Y_{1}}{⪰}^{STM}L_{Y_{2}}$, provided that either $W_{1}=W_{2}=\emptyset$ or the inequality $\inf_{v\in W_{1}}h_{V}\left(v\right)\geq \sup_{v\in W_{2}}h_{V}\left(v\right)$ is satisfied.*

*Note that the conditions in Remark 2 hold.*

**Proof.**  1. Given that $Y_{1}{⪰}^{ODIS}Y_{2}$, we may infer from Equation (24)

$$\begin{array}{cc} \left(\tau_{1}-\tau_{2}\right)\left(S\Lambda \tau_{1},\tau_{2}\left(L_{Y_{1}}\right)-S\Lambda \tau_{1},\tau_{2}\left(L_{Y_{2}}\right)\right)= & \int_{0}^{1}h_{V}^{\tau_{1}}\left(v\right)\left(k_{1}^{\tau_{1}-1}\left(K_{1}^{-1}\left(v\right)\right)-k_{2}^{\tau_{1}-1}\left(K_{2}^{-1}\left(v\right)\right)\right)dv\\ & -\int_{0}^{1}h_{V}^{\tau_{2}}\left(v\right)\left(k_{1}^{\tau_{2}-1}\left(K_{1}^{-1}\left(v\right)\right)-k_{2}^{\tau_{2}-1}\left(K_{2}^{-1}\left(v\right)\right)\right)dv\\ & \geq 0, \end{array}$$

where $\tau_{1}-\tau_{2}>1$, which leads to the desired result.

2. Given that $Y_{1}{⪰}^{STM}Y_{2}$, it follows from Equation (24) that when $\tau_{1}-\tau_{2}>1$, we obtain

(29) $$\begin{array}{c} \int_{0}^{1}\left(k_{1}^{\tau_{1}-1}\left(K_{1}^{-1}\left(v\right)\right)-k_{2}^{\tau_{1}-1}\left(K_{2}^{-1}\left(v\right)\right)\right)dv-\int_{0}^{1}\left(k_{1}^{\tau_{2}-1}\left(K_{1}^{-1}\left(v\right)\right)-k_{2}^{\tau_{2}-1}\left(K_{2}^{-1}\left(v\right)\right)\right)dv\geq 0. \end{array}$$

The follow-up gives us

$$\begin{array}{cc} \left(\tau_{1}-\tau_{2}\right)\left(S\Lambda \tau_{1},\tau_{2}\left(L_{Y_{1}}\right)-S\Lambda \tau_{1},\tau_{2}\left(L_{Y_{2}}\right)\right)= & \int_{0}^{1}h_{V}^{\tau_{1}}\left(v\right)\left(k_{1}^{\tau_{1}-1}\left(K_{1}^{-1}\left(v\right)\right)-k_{2}^{\tau_{1}-1}\left(K_{2}^{-1}\left(v\right)\right)\right)dv\\ & -\int_{0}^{1}h_{V}^{\tau_{2}}\left(v\right)\left(k_{1}^{\tau_{2}-1}\left(K_{1}^{-1}\left(v\right)\right)-k_{2}^{\tau_{2}-1}\left(K_{2}^{-1}\left(v\right)\right)\right)dv \end{array}$$

Therefore, by utilizing (29) along with the condition $\inf_{v\in W_{1}}h_{V}\left(v\right)\geq \sup_{v\in W_{2}}h_{V}\left(v\right)$ for $\tau_{1}-\tau_{2}>1$, we arrive at

$$\begin{array}{cc} \; & \int_{W_{1}}h_{V}^{\tau_{1}}\left(v\right)\left(k_{1}^{\tau_{1}-1}\left(K_{1}^{-1}\left(v\right)\right)-k_{2}^{\tau_{1}-1}\left(K_{2}^{-1}\left(v\right)\right)\right)dv\\ & +\int_{W_{2}}h_{V}^{\tau_{1}}\left(v\right)\left(k_{1}^{\tau_{1}-1}\left(K_{1}^{-1}\left(v\right)\right)-k_{2}^{\tau_{1}-1}\left(K_{2}^{-1}\left(v\right)\right)\right)dv\\ & -\int_{W_{1}}h_{V}^{\tau_{2}}\left(v\right)\left(k_{1}^{\tau_{2}-1}\left(K_{1}^{-1}\left(v\right)\right)-k_{2}^{\tau_{2}-1}\left(K_{2}^{-1}\left(v\right)\right)\right)dv\\ & -\int_{W_{2}}h_{V}^{\tau_{2}}\left(v\right)\left(k_{1}^{\tau_{2}-1}\left(K_{1}^{-1}\left(v\right)\right)-k_{2}^{\tau_{2}-1}\left(K_{2}^{-1}\left(v\right)\right)\right)dv\\ & \geq {\left(\underset{v\in W_{1}}{\inf}h_{V}\left(v\right)\right)}^{\tau_{1}}\int_{W_{1}}\left(k_{1}^{\tau_{1}-1}\left(K_{1}^{-1}\left(v\right)\right)-k_{2}^{\tau_{1}-1}\left(K_{2}^{-1}\left(v\right)\right)\right)dv\\ & +{\left(\underset{v\in W_{2}}{\sup}h_{V}\left(v\right)\right)}^{\tau_{1}}\int_{W_{2}}\left(k_{1}^{\tau_{1}-1}\left(K_{1}^{-1}\left(v\right)\right)-k_{2}^{\tau_{1}-1}\left(K_{2}^{-1}\left(v\right)\right)\right)dv\\ & -{\left(\underset{v\in W_{1}}{\inf}h_{V}\left(v\right)\right)}^{\tau_{2}}\int_{W_{1}}\left(k_{1}^{\tau_{2}-1}\left(K_{1}^{-1}\left(v\right)\right)-k_{2}^{\tau_{2}-1}\left(K_{2}^{-1}\left(v\right)\right)\right)dv\\ & -{\left(\underset{v\in W_{2}}{\sup}h_{V}\left(v\right)\right)}^{\tau_{2}}\int_{W_{2}}\left(k_{1}^{\tau_{2}-1}\left(K_{1}^{-1}\left(v\right)\right)-k_{2}^{\tau_{2}-1}\left(K_{2}^{-1}\left(v\right)\right)\right)dv\\ & \geq {\left(\underset{v\in W_{2}}{\sup}h_{V}\left(v\right)\right)}^{\tau_{1}}\int_{W_{1}}\left(k_{1}^{\tau_{1}-1}\left(K_{1}^{-1}\left(v\right)\right)-k_{2}^{\tau_{1}-1}\left(K_{2}^{-1}\left(v\right)\right)\right)dv\\ & +{\left(\underset{v\in W_{2}}{\sup}h_{V}\left(v\right)\right)}^{\tau_{1}}\int_{W_{2}}\left(k_{1}^{\tau_{1}-1}\left(K_{1}^{-1}\left(v\right)\right)-k_{2}^{\tau_{1}-1}\left(K_{2}^{-1}\left(v\right)\right)\right)dv\\ & -{\left(\underset{v\in W_{2}}{\sup}h_{V}\left(v\right)\right)}^{\tau_{2}}\int_{W_{1}}\left(k_{1}^{\tau_{2}-1}\left(K_{1}^{-1}\left(v\right)\right)-k_{2}^{\tau_{2}-1}\left(K_{2}^{-1}\left(v\right)\right)\right)dv\\ & -{\left(\underset{v\in W_{2}}{\sup}h_{V}\left(v\right)\right)}^{\tau_{2}}\int_{W_{2}}\left(k_{1}^{\tau_{2}-1}\left(K_{1}^{-1}\left(v\right)\right)-k_{2}^{\tau_{2}-1}\left(K_{2}^{-1}\left(v\right)\right)\right)dv\\ & ={\left(\underset{v\in W_{2}}{\sup}h_{V}\left(v\right)\right)}^{\tau_{1}}\int_{0}^{1}\left(k_{1}^{\tau_{1}-1}\left(K_{1}^{-1}\left(v\right)\right)-k_{2}^{\tau_{1}-1}\left(K_{2}^{-1}\left(v\right)\right)\right)dv\\ & -{\left(\underset{v\in W_{2}}{\sup}h_{V}\left(v\right)\right)}^{\tau_{2}}\int_{0}^{1}\left(k_{1}^{\tau_{2}-1}\left(K_{1}^{-1}\left(v\right)\right)-k_{2}^{\tau_{2}-1}\left(K_{2}^{-1}\left(v\right)\right)\right)dv\geq 0. \end{array}$$

□

The following theorem shows that the shortest lifetime in the equal and independent case has a lower or equal Sharma–Taneja–Mittal entropy order than for all of the mixed systems in the setting of the decreasing failure rate of each lifetime component.

**Theorem** **13.** *Assume that the lifespan component has a declining failure rate and that the cases are equal and independent. Then, for the mixed lifetime mechanism denoted by L, we have $Y_{1:p}{⪰}^{STM}L$. Note that the conditions in Remark 2 hold.*

**Proof.**  As stated by Bagai and Kochar [26], when the lifetime has a decreasing failure rate, it holds that $Y_{1:p}{⪰}^{OHR}L$ implies $Y_{1:p}{⪰}^{ODIS}L$. From Theorem 10, we then conclude that $Y_{1:p}{⪰}^{STM}L$. □

**Theorem** **14.** *Given the assumption that $S\Lambda \tau_{1},\tau_{2}\left(Y_{r:p}\right)<\infty$, we obtain from Equation (27)*

$$\begin{array}{c} S\Lambda \tau_{1},\tau_{2}\left(L\right)\leq \sum_{r=1}^{p}\delta_{r}S\Lambda \tau_{1},\tau_{2}\left(Y_{r:p}\right), \end{array}$$

*where $S\Lambda \tau_{1},\tau_{2}\left(Y_{r:p}\right)$ is the Sharma–Taneja–Mittal entropy of the r-th order statistic. Note that the conditions in Remark 2 hold.*

**Proof.**  Referring back to Equation (28), we obtain

$$\begin{array}{cc} S\Lambda \tau_{1},\tau_{2}\left(L\right) & =\frac{1}{\tau_{1}-\tau_{2}}\left(\int_{0}^{\infty }{\left(\sum_{r=1}^{p}\delta_{r}k_{r:p}\left(l\right)\right)}^{\tau_{1}}dl-\int_{0}^{\infty }{\left(\sum_{r=1}^{p}\delta_{r}k_{r:p}\left(l\right)\right)}^{\tau_{2}}dl\right). \end{array}$$

When we apply Jensen’s inequality, we obtain

$$\begin{array}{c} {\left(\sum_{r=1}^{p}\delta_{r}k_{r:p}\left(l\right)\right)}^{\tau_{i}}\leq \sum_{r=1}^{p}\delta_{r}k_{r:p}^{\tau_{i}}\left(l\right), \end{array}$$

where $k_{r:p}^{\tau_{i}}$ exhibits convexity when $\tau_{i}>1$ and l>0, with i=1,2. Therefore,

$$\begin{array}{c} \int_{0}^{\infty }k_{L}^{\tau_{i}}\left(l\right)dl=\int_{0}^{\infty }{\left(\sum_{r=1}^{p}\delta_{r}k_{r:p}\left(l\right)\right)}^{\tau_{i}}dl\leq \sum_{r=1}^{p}\delta_{r}\int_{0}^{\infty }k_{r:p}^{\tau_{i}}\left(l\right)dl, \end{array}$$

(30) $$\begin{array}{c} ⟹\int_{0}^{\infty }{\left(\sum_{r=1}^{p}\delta_{r}k_{r:p}\left(l\right)\right)}^{\tau_{i}}dl\leq \sum_{r=1}^{p}\delta_{r}\int_{0}^{\infty }k_{r:p}^{\tau_{i}}\left(l\right)dl; \end{array}$$

by multiplying both sides of Equation (30) by $\frac{1}{\tau_{1}-\tau_{2}}$ and observing that $\tau_{1}-\tau_{2}>1$, the following holds:

$$\begin{array}{cc} S\Lambda \tau_{1},\tau_{2}\left(L\right) & \leq \frac{1}{\tau_{1}-\tau_{2}}\left[\sum_{r=1}^{p}\delta_{r}\int_{0}^{\infty }k_{r:p}^{\tau_{1}}\left(l\right)dl-\sum_{r=1}^{p}\delta_{r}\int_{0}^{\infty }k_{r:p}^{\tau_{2}}\left(l\right)dl\right]\\ & =\frac{1}{\tau_{1}-\tau_{2}}\left[\sum_{r=1}^{p}\delta_{r}\int_{0}^{\infty }k_{r:p}^{\tau_{1}}\left(l\right)dl-\sum_{r=1}^{p}\delta_{r}\int_{0}^{\infty }k_{r:p}^{\tau_{2}}\left(l\right)dl\right]\\ & =\sum_{r=1}^{p}\delta_{r}\left[\frac{1}{\tau_{1}-\tau_{2}}\left(\int_{0}^{\infty }k_{r:p}^{\tau_{1}}\left(l\right)dl-\int_{0}^{\infty }k_{r:p}^{\tau_{2}}\left(l\right)dl\right)\right]\\ & =\sum_{r=1}^{p}\delta_{r}S\Lambda \tau_{1},\tau_{2}\left(Y_{r:p}\right). \end{array}$$

□

## 6. Conclusions

This study explores additional properties of Sharma–Taneja–Mittal entropy and its related measures. A new non-parametric estimation method for residual cumulative Sharma–Taneja–Mittal entropy, based on the U-statistic, is presented. The efficiency of this estimator is demonstrated through a simulation study, which also examines its sensitivity to the values of $\tau_{1}$ and $\tau_{2}$. Moreover, we investigate the non-positivity, stochastic comparisons, and connections of dynamic residual cumulative Sharma–Taneja–Mittal entropy with the hazard rate function and mean residual function. Additionally, we establish bounds for this measure and examine its relationship with equilibrium random variables. Furthermore, an alternative dynamic form of residual cumulative Sharma–Taneja–Mittal entropy is introduced, along with its properties in the context of record values. Notably, this alternative form does not always guarantee negativity. Finally, we analyze the Sharma–Taneja–Mittal entropy of coherent systems, providing bounds and conducting stochastic comparisons. In future work, we aim to conduct a detailed investigation of the alternative dynamic measure, including the identification of distributional classes under which it retains desirable properties and a clearer delineation of the conditions in which it provides meaningful and stable uncertainty quantification. Moreover, we can discuss the relationships between Sharma–Taneja–Mittal entropy and other entropies (Shannon entropy, Kaniadakis entropy, Rényi entropy), see [27,28,29,30,31,32,33].

## Figures and Tables

**Table 1:** The variance and MSER of the residual cumulative Sharma–Taneja–Mittal entropy estimator using the exponential distribution with rate 0.5.

$\tau_{1},\tau_{2}$	$RS\Lambda_{\tau_{1},\tau_{2}}\left(Y\right)$	p=10		p=20		p=30	
		Variance	MSER	Variance	MSER	Variance	MSER
1, 2	−1	0.1411084	0.3756253	0.06803551	0.260849	0.04363882	0.2088998
1, 3	−0.666667	0.05668502	0.2380988	0.02708766	0.1645907	0.01740624	0.1319349
2, 3	−0.333333	0.01562275	0.1249857	0.007080991	0.08414882	0.004543261	0.0674062
2, 4	−0.25	0.008945401	0.09457654	0.004069401	0.06379144	0.002620203	0.05119015
$\tau_{1},\tau_{2}$	$RS\Lambda_{\tau_{1},\tau_{2}}\left(Y\right)$	p=50		p=70		p=100	
		Variance	MSER	Variance	MSER	Variance	MSER
1, 2	−1	0.02679797	0.1636931	0.01918941	0.138519	0.01334832	0.1155292
1, 3	−0.666667	0.01069409	0.1034071	0.007683631	0.08765201	0.005359265	0.07320337
2, 3	−0.333333	0.002719683	0.05214846	0.001949153	0.04414716	0.001366653	0.03696643
2, 4	−0.25	0.001555784	0.03944218	0.001114206	0.03337828	0.0007832065	0.02798447

**Table 2:** The variance and MSER of the residual cumulative Sharma–Taneja–Mittal entropy estimator using Gamma(2,2) distribution.

$\tau_{1},\tau_{2}$	$RS\Lambda_{\tau_{1},\tau_{2}}\left(Y\right)$	p=10		p=20		p=30	
		Variance	MSER	Variance	MSER	Variance	MSER
1, 2	−1.5	0.2246712	0.4740772	0.1049496	0.3239506	0.06833788	0.2614115
1, 3	−1.03704	0.09447327	0.3074325	0.04419804	0.2102305	0.02873103	0.1694998
2, 3	−0.574074	0.03160255	0.1778131	0.01409945	0.1187435	0.009015945	0.09494975
2, 4	−0.445313	0.01957194	0.1399292	0.008637679	0.09294131	0.005517741	0.07427972
$\tau_{1},\tau_{2}$	$RS\Lambda_{\tau_{1},\tau_{2}}\left(Y\right)$	p=50		p=70		p=100	
		Variance	MSER	Variance	MSER	Variance	MSER
1, 2	−1.5	0.04144159	0.2035619	0.02979252	0.1726007	0.02055914	0.1433913
1, 3	−1.03704	0.01746553	0.1321506	0.01255328	0.1120405	0.008621775	0.09285993
2, 3	−0.574074	0.005342667	0.07309	0.003807901	0.06171099	0.002601418	0.05100904
2, 4	−0.445313	0.003255807	0.05705699	0.002307225	0.04803638	0.001577914	0.039727

**Table 3:** Bootstrap estimates of the proposed estimator for different $\left(\tau_{1},\tau_{2}\right)$ combinations based on 10,000 resamples.

$\tau_{1}$	$\tau_{2}$	Original	Mean	SD	Bias	95% CI (Lower)	95% CI (Upper)
1	2	−40.5905	−38.0535	14.9829	2.5370	−65.4670	−7.8855
1	3	−24.0000	−23.2474	9.8673	0.7527	−43.8650	−5.7365
2	3	−7.4095	−8.4571	5.2065	−1.0475	−21.9330	−2.2886
2	4	−4.8996	−5.7273	3.5175	−0.8277	−15.0670	−1.6549

## Data Availability

All datasets analyzed or generated during this study are included within the article.
